# Health Tract, No. 76

**Published:** 1862-06

**Authors:** 


HEALTH TRACT, No. 76.

### A CENTENARIAN.

WE have just received a letter from "HUGH CULL," who was born at Havre de Grace, Maryland, in October, seventeen hundred and fifty-seven. In 1760 he went to the "West," which was then in Pennsylvania; twenty years later, he went to the "West" again, which by that time had moved itself to Lexington, Kentucky; a quarter of a century further on, he followed the "West," and found it in Wayne county, Indiana, where he has lived ever since. For three quarters of a century he has been a member of the Methodist Church, and for fifty-five years one of its ministers; "never wrote a sermon in my life, never used notes." His "circuit" was two hundred miles long and twenty broad, made in four weeks, preaching every day. Father Cull is five feet ten, weighs one hundred and sixty pounds, heavy eyebrows, and very black hair, never having changed its color. For the first fifty years of his life his chief diet was corn-bread and sweet milk. He never could eat any thing acid, not even a sour apple. His abstinence from any kind of food was not with reference to its healthfulness, but solely because he had no relish for it, hence does not eat boiled "victuals," and takes no vegetables or fresh meat. He is a very little eater, not fond of a variety. A little salt meat, bread, and very sweet coffee, constitute his main diet. He takes no stimulants, (liquor,) never spent a dollar for whisky; yet chews tobacco, "and always has." He sleeps more than almost any other man, goes to bed at dark and rises with the sun, winter and summer, always taking his breakfast late. Has worked on his farm for a living, never worked hard, but in moderation; is very confident that he never had any children—whether he had a wife, does not say. His sermons are always short and earnest. If he will only re-emigrate, we will promise him one of the largest and most *appreciative* congregations in New-York. He has lived in the same house fifty-two years. He has a cheerful disposition, and evidently is of an inquiring turn of mind, for in a postscript he wishes to know who baptized John the Baptist. No doubt this question has been "pestering" him for a hundred years. Will some of our double D'd readers vouchsafe the long desired information, and thus enable him to die with one less "weight on his mind?" The practical inquiry is, to what does this Father in Israel owe his great age and healthfulness? Plainly, to a simple mode of life; the moderate eating of such plain food as was agreeable to his taste; habitual, moderate daily labor on a farm, and an earnest industry in doing good to others, and striving to get better himself.

NEW-YORK, April 20th, 1862.

